# Effect of sequential treatment with syndrome differentiation on acute exacerbation of chronic obstructive pulmonary disease and "AECOPD Risk-Window": study protocol for a randomized placebo-controlled trial

**DOI:** 10.1186/1745-6215-13-40

**Published:** 2012-04-20

**Authors:** Wang Haifeng, Li Jiansheng, Li Suyun, Yu Xueqing, Zhang Hailong, Wang Zhiwan, Wu Qiyi, Zhang Pankui, Wang Zhongchao, Li Fenglei, Yan Haihong

**Affiliations:** 1 Department of Respiratory Diseases, The First Affiliated Hospital of Henan University of Traditional Chinese Medicine, Zhengzhou, Henan Province 450000, People's Republic of China; 2 Institute of Geriatrics, Henan University of Traditional Chinese Medicine, Zhengzhou, Henan Province 450008, People's Republic of China

**Keywords:** Chronic obstructive pulmonary disease, Exacerbations, Traditional Chinese Medicine, Treatment, Clinical trials

## Abstract

**Background:**

Frequent chronic obstructive pulmonary disease (COPD) exacerbation is a major cause of hospital admission and mortality. It has been reported that Traditional Chinese Medicine (TCM) may relieve COPD symptoms and reduce the incidence of COPD exacerbations, thus improving life quality of COPD patients. The acute exacerbation of COPD risk-window (AECOPD-RW) is the period after an exacerbation and before the patient returns to baseline. In the AECOPD-RW, patients are usually at increased risk of a second exacerbation, which may lead to hospital admission and high mortality. It may be beneficial for acute exacerbation of chronic obstructive pulmonary disease (AECOPD) patients to receive interventions during AECOPD-RW. During exacerbations the treatment principle is to eliminate exogenous pathogens, whereas the AECOPD-RW treatment principle focuses on enhancing body resistance.

**Methods/Design:**

A prospective, multi-center, single-blinded, double-dummy and randomized controlled clinical trial is being conducted to test the therapeutic effects of a sequential two stage treatment, which includes eliminating pathogen and strengthening vital qi with syndrome differentiation. A total of 364 patients will be enrolled in this study with 182 in each treatment group (TCM and control). Patients received medication (or control) according to their assigned group. TCM for AECOPD were administered twice daily to patients with AECOPD over 7 to 21 days, followed by TCM for AECOPD-RW over 28 days. All patients were followed for six months. The clinical symptoms, the modified medical research council dyspnea (MMRC) scale and exacerbations were used as the primary outcome measures. Pulmonary function, quality of life and mortality rate were used as secondary outcome measures.

**Discussion:**

It is hypothesized that sequentially eliminating pathogens and strengthening vital qi treatments with syndrome differentiation will have beneficial effects on reducing the frequency and duration of acute exacerbation, relieving symptoms and improving quality of life for COPD patients.

**Trial registration:**

This study is registered at ClinicalTrials.gov, ChiCTR-TRC-11001460.

## Background

Chronic obstructive pulmonary disease (COPD) is characterized by acceleration in the normal decline in lung function with age and by repeated exacerbations. These exacerbations are associated with worsening symptoms and lung function [1]. The frequency of exacerbation has been shown to be an important determinant of the impaired health-related quality of life seen in COPD patients [2] and to affect decline in lung function [3]. Exacerbations are a frequent cause of physician consultation in primary and secondary care and a major cause of hospital admission and death [4]. Therefore, the management of exacerbations places a considerable burden on the health services both in terms of physician consultation time and healthcare cost [5]. A reduction in exacerbation frequency would have a number of benefits for patients and health services alike [6]. Therefore, preventing exacerbations is a key treatment goal.

The global initiative for chronic obstructive lung diseases (GOLD) document suggests a follow-up at four to six weeks after a hospitalized exacerbation [7]. However, there is evidence that exacerbations tend to cluster in time, with a high-risk period of recurrence during the eight weeks following the first exacerbation [8]. This has important implications for the target of preventative interventions. Therefore, from our clinical observation, we have identified an acute exacerbation of chronic obstructive pulmonary disease risk-window (AECOPD-RW); that is the period after a first exacerbation and before the patient has returned to baseline. During this period, although major symptoms have been relieved, lung function has not gone back to stable baseline, and inflammation may also continue. Therefore, patients may be at increased risk of a second exacerbation, leading to hospital admission and a high mortality rate. Thus, it seems appropriate to intervene in the AECOPD-RW.

Traditional Chinese medicine (TCM) is commonly used for COPD in Asia. It has long been known that TCM is effective in relieving symptoms, reducing the incidence of COPD exacerbations and improving quality of life in COPD patients.

Our previous study showed that in an exacerbation period the treatment principle was to eliminate exogenous pathogens. When the patient enters the AECOPD-RW, the treatment principle should be supporting body resistance [9].

Our hypothesis was that a sequential TCM treatment aimed at eliminating pathogens and strengthening vital qi with syndrome differentiation may reduce exacerbation frequency in patients with AECOPD. In this paper, we report the protocol on a multi-center, randomized, single-blind, placebo-controlled trial to test this hypothesis.

### Objective

The objective is to determine whether sequentially eliminating pathogens and strengthening vital qi treatment with syndrome differentiation reduces COPD exacerbation frequency.

## Methods and design

### Study design

**Figure 1:**
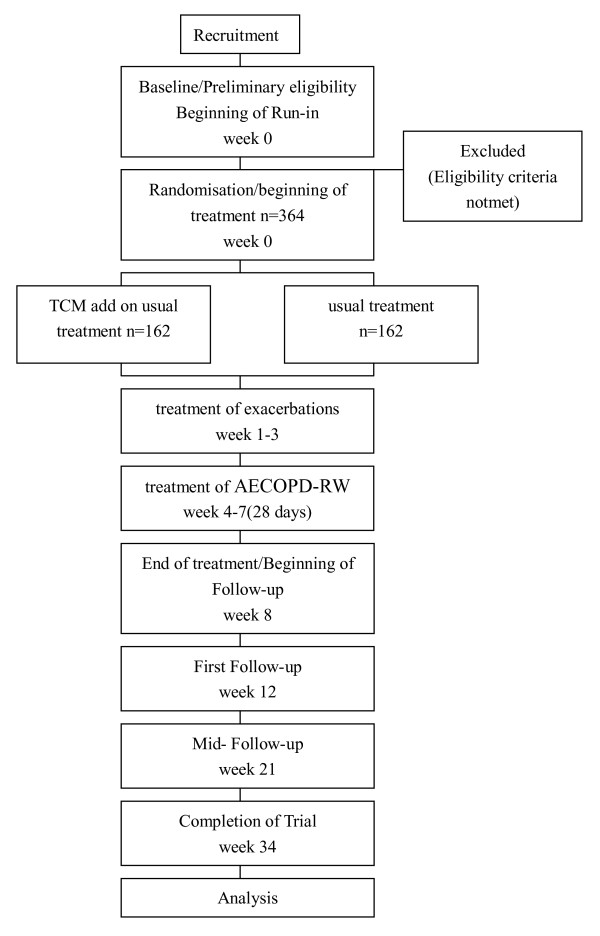
**Participation flow chart**.

This clinical trial is multi-centered, randomized, single-blind and placebo-controlled. The design of the study integrates rigorous, contemporary clinical research methodology in accord with principles set out in the Declaration of Helsinki and the Good Clinical Practice guidelines regarding the appropriate use of TCM in clinical practice. Reporting will be guided by the CONSORT statement [10,11]. Subjects are being enrolled at eight hospitals in four sites in China: 1) The First Affiliated Hospital of Henan University of TCM; 2) TCM hospital of Xinjiang Uygur Autonomous Region; 3) Sichuan Province TCM hospital; 4) Jiangsu Province Hospital of TCM; 5) the First Affiliated Hospital of Henan University of Science and Technology; 6) Henan University of Huaihe hospital; 7) The First Affiliated Hospital of Zhengzhou University; and 8) Henan Provincial Chest Hospital. The eight hospitals in China will randomly assign 364 patients with AECOPD in a ratio of 1:1 to the TCM treatment group or matching placebo. Consenting eligible participants will be enrolled for five to eight weeks and required to attend 12 visits in total. The trial has three phases: one to three weeks treatment with either TCM for AECOPD or placebo granules, four weeks treatment with either TCM for AECOPD-RW or placebo granules, and six months follow-up (Figure 1).

### Study duration

Ongoing recruitment will occur for a maximum period of 18 months (between July 2011 and December 2012) or until 364 individuals are randomized.

### Subjects

A total of 364 participants will be recruited through local advertising and doctor referrals from hospital outpatients and general practice clinics. Interested participants can telephone or email the trial coordinators at the corresponding sites for further information. Participant information and consent forms will be sent to interested individuals to be read prior to scheduling their first visit.

### Inclusion criteria for participation in the trial

The inclusion criteria are as follows: 1) males and females aged 40 to 80 years inclusive; 2) they must satisfy the COPD diagnostic criteria defined by GOLD (post-bronchodilator spirometry, forced expiratory volume in 1 second (FEV_1_)/forced vital capacity (FVC) < 0.7 and predicted FEV_1 _50% to 80%); 3) they must experience an acute infective exacerbation of COPD; 5) meet the TCM diagnostic criteria (syndrome of exogenous cold-evil and internalization, syndrome of accumulation of phlegm-dampness in the lung, syndrome of phlegm-heat obstructing in the lung); and 6) give written informed consent to participate.

### Exclusion criteria

Exclusion criteria include: 1) pregnancy, breast-feeding or women intending to become pregnant during the course of the study; 2) inability to understand the nature, scope and possible consequences of the study; 3) diagnosis of bronchiectasis or active tuberculosis, obliterative bronchiolitis or diffuse pantothcnic bronchiolitis, pneumothorax, pleural effusion or pulmonary embolism; 4) a history of bronchial asthma or neuromuscular disorder that affects respiration; 5) cancer; 6) respiratory failure that needs mechanical ventilation; 7) serious illnesses, such as heart, liver or kidney diseases, or unstable hemodynamics; 8) inability to adequately perform spirometry tests; 9) need for long periods of bed rest; 10) on long-term immunosuppressive agents or immunostimulants; 11) an allergic history to TCM products or participating in other trials.

This study will be conducted in accordance with protection of patients, as outlined in the Declaration of Helsinki. Each participant will sign the written informed consent before undergoing any examination or study procedure, in compliance with Good Clinical Practice. Patients whose syndrome differentiation of TCM is the syndrome of exogenous cold-evil and internalization, syndrome of accumulation of phlegm-dampness in the lung or syndrome of phlegm-heat obstructing in the lung are included. Patients who initially meet eligibility criteria will complete the additional baseline testing and will be randomized into either the treatment or the control group.

### Ethics issue

This study has been approved by the Ethics Committee of The First Affiliated Hospital of Henan University of TCM (No: YFYKYLL2011-001). Each participating center obtained a local Institutional Review Board Approval. All study participants will sign the written informed consent prior to participation.

### Interventions

Eligible patients will be randomized to one of the two arms: placebo or Chinese medical herb (11.65 g twice daily). Three TCM formulas for exacerbation will be administered orally for 7 to 21 days: sanhanhuayinfang (*Ephedra, Rumulus Ginnamomi, Rhizoma Zingiberis*, and so on), qingrehuatanfang (*Mongolian snakegourd fruit, Rhizoma Pinelliae, Bulbus Fritillariae Cirrhosae *and so on), zaoshihuatanfang (*Prepared Pinellia Tuber, Officinal Magnolia Bark, Tangerine Peel *and so on). There are also three TCM formulas for AECOPD-RW that will be administered orally for four weeks; they are: bufeijianpifang (*Codonopsis, Astragalus root, Largehead Atractylodes Rhizome, Perilla Fruit, Mangnolia officinalis*, and so on), bufeiyishenfang (*Ginseng, Astragalus root, medlar *and so on), yiqizishenfang (*Ginseng, solomonseal, Rehmannia Glutinosa *and so on). All drugs were made into granules by Jiangyin Tianjiang Pharmaceutical Co., Ltd. Jiangyin city, Jiangsu province, China). In this study, the subjects and statisticians will be blinded to treatment assignment. In this respect, the trial is single-blind. Randomization of subjects will occur centrally using a random number generator and will be stratified by syndrome differentiation of TCM. (Syndrome, as related to illnesses in Western medicine, is composed of a set of signs and/or symptoms classified by Traditional Chinese Medicine practitioners. TCM Syndrome differentiation is based on symptoms and helps to identify a subset of disease). Patients will visit the doctor at one-, three- and six-month follow-ups.

### Randomization and allocation

Treatment allocation occurs when the study participant meets the inclusion criteria and signs the informed consent form. The teletherapist will then register the participant into the database, which, in turn, asks if they are ready to be randomized. After the teletherapist enters Yes, the site-specific randomization program displays the participant's group assignment number (placebo versus Chinese medical herb). Site-specific randomization lists will be computer-generated (that is, generated by an individualized basic visual code program) and concealed from the researchers by a senior data manager, who is not involved in the study. This information will remain confidential and is not shared with the study sites, in concordance with the CONSORT guidelines. This trial uses a prospective, randomized, outcome-blinded design, in which all outcome assessments are made by a research assistant blinded to treatment allocation and uninvolved in patient consent and management. The allocation list will be protected by password access files and held by an independent non-investigator. In the event of a clinical emergency, the individual's randomization code and group allocation can be identified.

### Sample size

The sample size calculation is based on the effect size of clinical symptom changes in AECOPD subjects from a previous trial of one of the most commonly used TCM injections [12]. In that study, the mean effective change in clinical symptoms was 97% in the treatment group, compared to 87% in the placebo group (*P *< 0.05). For the proposed study, to achieve a similar difference between the TCM and placebo treatment groups with an 90% power and a two-tailed significance level of 5%, *n*_1 _= *n*_2 _= 1,641.6 × [[]]^2^, the sample size in each group is 145. Considering a 20% drop-out rate over the course of the study, 182 patients will be enrolled in each group, that is, 364 in total. Intention-to-treat analysis will be applied to minimize bias due to drop-outs.

### Outcome measurements

#### Primary outcome measure

##### Exacerbations

Frequency, nature and severity of exacerbations of COPD will be evaluated in the first, the third and the sixth month during the follow-up period. If the interval between two events of acute exacerbation is within five days, it can be counted as one acute exacerbation.

Respiratory symptoms are classified as "major" symptoms (dyspnea, sputum purulence, sputum amount) or "minor" symptoms (wheezing, sore throat, cough, and symptoms of a common cold which were nasal congestion/discharge). Exacerbations are defined as the presence for at least two consecutive days of an increase in any two "major" symptoms or an increase in one "major" and one "minor" symptom according to criteria modified from Anthonisen and colleagues [13]. The first of the two consecutive days is taken as the day of onset of exacerbation.

We will also analyze recovery from exacerbation, because in most clinical studies a patient not having an exacerbation for four to six weeks would be considered stable. The time to the onset of the next exacerbation is defined as the number of days from onset of one exacerbation to onset of the next exacerbation [1].

### Symptoms and signs

Symptoms including cough, sputum, dyspnea, fever, acratia, cyanosis, wheezing and respiratory rate, and so on, will be evaluated on days 0 (admission), 4, 7, 14 and 21 during the exacerbation period, each week during the AECOPD-RW period, and in the first, the third and the sixth month during the follow-up period.

### Dyspnea

The modified medical research council dyspnea (MMRC) scale, which was first developed by the British Medical Research Council (MRC), and later revised by the American Thoracic Society (MMRC), will be observed and recorded before treatment, each week during the treatment period, and in the first, the third and the sixth month during the follow-up period.

### Secondary outcome measure

#### Secondary outcome measures include efficacy and safety components

2.1 Quality of life (QoL): QoL assessed by the COPD Assessment Test (CAT) will be observed and recorded on Day 0 (admission), after exacerbation and after AECOPD-RW during the treatment period, and in the first, the third and the sixth month during the follow-up period.

2.2 Lung function: Lung function using spirometry indices of FEV_1_, FVC and FEV1/FVC ratio will be tested and recorded on Day 0 (admission), after exacerbation and after AECOPD-RW during the treatment period, and in the third month during the follow-up period.

2.3 Mortality rate: All-cause mortality will be recorded at the end of the sixth month during the follow-up period.

### Adverse event reporting

Routine calibration and standard operating procedures will be uniformly undertaken at each site. Participants will be advised not to use respiratory medications for several hours prior to their spirometry testing. Two sets of measurements will be taken at each visit, pre-bronchodilator and post-bronchodilator (post 400 μg of inhaled Salbutamol).

Any adverse events will be listed in the trial record and followed up to completion by the trial coordinators and respiratory physicians. Adverse event details will be scored using a six-point scale, 0 = none, 1 = minimal, 2 = mild, 3 = moderate, 4 = severe, and 5 = extremely severe. Participants will be able to report adverse events anytime throughout the trial and will receive advice accordingly. Serious adverse events will be reported to the reviewing HREC and site HRECs within the timeframe specified by the lead HREC.

Throughout the study period trial coordinators will contact the participant's usual treating doctor to record relevant data on presentations and exacerbations.

To assist with outcome documentation and medication compliance, participants will be given a participant diary for the duration of the trial. They will be asked to record trial medication and relief medication usage as well as any new medications they commence during the trial.

Participants may withdraw from the study for any reason at any time without repercussion. They will only be withdrawn by investigators if it is deemed medically unsafe for them to continue. Dropouts will not be replaced.

### Statistical analysis

A descriptive statistical analysis will be performed for all study variables. We will calculate the mean, median and standard deviations for quantitative variables, and the absolute and relative frequency for qualitative variables.

We will use SPSS 19.0 (License number: 6f1d84c801f1e6010dc, SPSS Inc. Chicago, Illinois state, United States) to statistically analyze the data. An intention-to-treat analysis will be applied using the Last Observation Carried Forward (LOCF) method for missing data. Analysis of covariance with baseline as covariate will be used to assess differences in treatment outcomes between the two groups at each of these time points. To correct for inflated risk of Type 1 error, multiple comparison procedures suggested by Ludbrook et al. will be used [14]. The use of relief medication during the trial will be investigated for its effect on the outcome measures by using it as a covariate in the statistical analysis. A Data Safety Monitoring Board has been established to assess the progress of the trial, particularly safety endpoints.

## Discussion

Chronic Obstructive Pulmonary Disease (COPD) remains a major public health problem. Exacerbations of COPD are an important cause of the considerable morbidity and mortality found in COPD. The aim of AECOPD treatment should be to decrease symptoms and exacerbations, reduce the risk of hospital admission and increase lung function and improve QoL. Recently, the approach to therapy has focused on symptomatic relief.

Traditional Chinese Medicine (TCM), as a system of medicine, has been a form of health care in China and its neighboring countries for several thousand years. It has been reported that TCM therapy, such as tanreqing injection, could improve the Chinese medical signs and symptoms in patients with AECOPD and improve airway inflammation, airway mucus hypersecretion [15,16], and lung function and relieve airway inflammation in patients with stable COPD [17].

The recently updated "Global initiative for chronic Obstructive Lung Disease" guidelines recognize that after discharge from the hospital, care should provide for four- to six-week follow-up assessment, with an absence of exacerbation for four to six weeks being considered stable [1]. More recently, data have emerged that the timing of a second exacerbation after a first event is indeed nonrandom [8], and may relate to the therapy used. Therefore, we introduce the concept of the AECOPD risk-window (AECOPD-RW). That is, after a first exacerbation, before the patient returns to stable baseline, despite relief of major symptoms, poor lung function and inflammation may persist. In this period, patients may be at increased risk of a second exacerbation, which is responsible for hospital admission and increased mortality. It seems appropriate, therefore, to provide interventions for AECOPD-RW.

If patients are given TCM in addition to the usual treatment during the AECOPD-RW period, it may resolve COPD.

The study will be a large, multi-center, long-term trial of chronic obstructive pulmonary disease, and the data gathered will shed new light on traditional Chinese medicine for COPD treatment.

### Trial status

At the time of manuscript submission, we have recruited 105 patients but have not completed patient recruitment.

## Abbreviations

AECOPD-RW: Acute exacerbation of COPD risk-window; CAT: COPD assessment test; COPD: Chronic obstructive pulmonary disease; FEV1: Forced expiratory volume in 1 second; FVC: Forced vital capacity; GOLD: Global Initiative for Chronic Obstructive Lung Disease; LOCF: Last observation carried forward; MMRC: Modified Medical Research Council; QoL: Quality of life; RCT: Randomized controlled trial; TCM: Traditional Chinese Medicine.

## Competing interests

The authors declare that they have no competing interests.

## Authors' contributions

WH has been involved in drafting the manuscript and writing it. He has participated in the design of the study and in the intervention. LJ has been involved in the design of the study and he has participated in reviewing the manuscript. LS and YX have been involved in the design of the intervention and participated in reviewing the manuscript. ZH has been involved in the design of the study, and he has participated in reviewing the manuscript. WZ has been involved in drafting the manuscript and writing it. She has participated in the design of the study and the intervention. WQ, ZP, WZ, ZP, LF and YH have participated in reviewing the manuscript. All authors read and approved the final manuscript.
